# Perceived Supervisor Support and Academic Procrastination in Postgraduate Students: Roles of Basic Psychological Needs Satisfaction and Learning Engagement

**DOI:** 10.3390/bs14111005

**Published:** 2024-10-30

**Authors:** Lumeng Wang, Guoxia Wang

**Affiliations:** School of Psychology, Northeast Normal University, Changchun 130024, China; lennonwong7@163.com

**Keywords:** perceived supervisor support, academic procrastination, basic psychological needs satisfaction, learning engagement, postgraduate, self-determination theory, chain-mediating effect

## Abstract

Academic procrastination is a common problem among postgraduate students, one that has caused negative consequences that cannot be ignored. Therefore, finding out how to effectively eliminate academic procrastination behavior has become an essential task in educational practice. Based on the use of Self-Determination Theory (SDT) to explore the relationship between the perceived supervisor support and academic procrastination of postgraduate students, a total of 448 questionnaires were gathered from postgraduate students across China. The results showed significant correlations between the perceived supervisor support, basic psychological needs satisfaction, learning engagement, and academic procrastination of postgraduate students. The relationship between the perceived supervisor support and academic procrastination of postgraduate students was mediated by basic psychological needs satisfaction and learning engagement. Additionally, basic psychological needs satisfaction and learning engagement played a chain-mediating role between the perceived supervisor support and academic procrastination of postgraduate students. The findings of this study contributed to the enhancement of the interaction between supervisors and students in higher education and improving the quality of postgraduate education.

## 1. Introduction

In today’s educational environment, the phenomenon of academic procrastination is particularly widespread among postgraduate students. Compared with undergraduates, postgraduate students have fewer scheduled classes and more flexible time to schedule on their own, which increases the likelihood of academic procrastination [1,2]. Procrastination not only affects the quality of academic achievements but also aggravates the pressure of postgraduate students during their study, resulting in pain and anxiety [3,4,5,6]; reducing procrastination can enhance the quality of postgraduate students. Procrastination, defined as the unnecessary delay in the execution of tasks that causes anxiety, is extremely common among college students and is significantly associated with poor academic performance, possibly leading to lower grades and even dropout [7]. Based on this definition, this study defined academic procrastination of postgraduate students as the unnecessary delay in completing academic tasks of postgraduate students, and this behavior will cause students to feel uneasy. The causes of procrastination among postgraduate students are complicated and diverse. Related studies have demonstrated that both internal and external factors influence students’ procrastination. Internal factors include an individual’s self-efficacy, academic engagement, and self-control ability [4,5,8,9], while external factors include the use of social networks, teachers’ teaching styles, and the teacher–student relationship [3,10,11]. In particular, the supervisor, as an important other who interacts frequently with postgraduate students during their study, has a substantial impact on their academic attitudes, behaviors, and development in their chosen fields of academic study [12].

### 1.1. The Relationship Between Perceived Supervisor Support and Academic Procrastination of Postgraduate Students

Perceived supervisor support refers to the degree of support that postgraduate students experience from their supervisors regarding their three basic psychological needs: autonomy, competence, and relatedness during the supervisor–student interactions. When supervisors provide support in the above three needs, students can perceive that this support meets their basic psychological needs. For example, supervisors provide support through methods such as offering options, providing assistance, affirming students’ capabilities, and caring for and understanding students [13]. Currently, empirical research has explored the effects of teacher autonomy-supportive and teacher control-oriented teaching styles on students in the classroom environment. According to research findings, there is a negative correlation between the autonomy-supportive teaching style and academic procrastination among college students, while the control-oriented teaching style has a negative impact on students and may be associated with a higher degree of academic procrastination [14,15]. However, postgraduate students do not receive supervisor’s support from the classroom teaching, with it being more often provided in the forms of group meetings, email exchanges, and frequent one-on-one guidance, etc., for their project or thesis guidance. This small-scale communication-based supervisor teaching mode is different from the traditional classroom teaching mode. Based on the theory of Leader-Member Exchange (LMX) and the Social Cognitive Theory (SCT), a study has explored the group of postgraduate students by sampling subjects from several domestic universities, regarding supervisors as “leaders” and postgraduate students as “subordinates”. It was also found that supervisor support enabled students to respond more actively to academic challenges and reduce academic procrastination [3]. In contrast, we started from the perspective of Self-Determination Theory (SDT), regarded postgraduate students as independent individuals with autonomous decision-making abilities, and emphasized that supervisors and postgraduate students do not have a “superior–subordinate” relationship. In the supervisor–student relationship, there was no underlying obligation of “students needing to obey the supervisor’s arrangements”. In contrast, supervisors served as more of a guide in the supervisor–student interaction.

**Hypothesis 1.**  *The more that support from the supervisor is perceived, the less academic procrastination postgraduate students will have*.

### 1.2. The Mediating Effect of Basic Needs Satisfaction

Under the framework of SDT, basic psychological needs are regarded as the psychological resources necessary for individual adjustment, integration, and growth [16]. According to SDT, basic psychological needs can be classified into three primary categories: autonomy; competence; and relatedness needs, which are considered innate rather than acquired through learning [17]. When any of these fundamental needs are obstructed, it might adversely affect an individual’s motivation and mental health [18]. In the educational environment, when teachers’ behaviors meet students’ three basic psychological needs, students will feel satisfied. Conversely students will experience frustration in their psychological needs [17]. The correlation between basic psychological needs satisfaction and academic procrastination has been well established. Studies have demonstrated that when a student’s basic psychological needs are adequately satisfied, their tendency to procrastinate would decrease [15,19,20]. Furthermore, research on the mediating role of basic psychological needs satisfaction related to teacher support has demonstrated that basic psychological needs satisfaction played a significant mediating role between teacher support and academic burnout, as well as between teacher support and learning engagement [21,22]. Although these studies did not directly explore academic procrastination, there was evidence that academic burnout and academic engagement were correlated with academic procrastination [23,24,25,26,27,28]. These studies also supported the correlation between teacher support and basic psychological needs satisfaction. However, the majority of them focused on teachers’ support for students’ autonomy and neglected the importance of teachers’ support for other basic psychological needs. In conclusion, there is a lack of studies exploring the mediating effect of basic psychological needs satisfaction on perceived supervisor support and the academic procrastination of postgraduate students.

**Hypothesis 2.**  *Basic psychological needs satisfaction plays a mediating role between perceived supervisor support and postgraduates’ academic procrastination*.

### 1.3. The Mediating Effect of Learning Engagement

Learning engagement is the state of being fully and positively involved in the process of learning. It is characterized by vigor, dedication, and absorption. Such engagement does not come from emotional cognition in short periods or specific situations; instead, it is a state that is enduring and widespread [29]. Learning engagement, as the key internal factor affecting academic procrastination, should also be paid attention to and always play a crucial role in the development of students. Existing studies have revealed that teacher support is a key factor affecting learning engagement [30], and they pointed out that there was a significant positive correlation between perceived teacher support and learning engagement [30,31,32,33]. Liu et al. took 869 primary school students in China as subjects to explore the mechanism of teacher support on students’ math learning engagement, finding that teacher support had a direct positive influence on math learning engagement. The study of An et al. also confirmed that teacher support could promote junior high school students’ learning engagement, further verifying this view. In addition, the positive impact of learning engagement on academic procrastination has also been demonstrated by research. The research discovered that learning engagement had a strong buffer effect on the detrimental effects of academic procrastination. It can improve students’ learning efficiency, and there was a significant negative correlation between learning engagement and academic procrastination [9,27].

**Hypothesis 3.**  *Learning engagement plays a mediating role between perceived supervisor support and postgraduates’ academic procrastination*.

### 1.4. The Chain Mediating Effect of Basic Needs Satisfaction and Learning Engagement

Basic psychological needs satisfaction enhances learning engagement; conversely, when individuals feel frustration with basic psychological needs, learning disengagement occurs [34]. We hypothesized that the satisfaction of basic psychological needs would have a significant impact on the learning engagement of postgraduate students. Through the review of the previous literature, we found that teacher support plays an important role in satisfying students’ basic psychological needs [21,22]; when students’ basic psychological needs are satisfied, they will be more inclined to devote themselves to their studies [34], which predicts their academic procrastination [9,23,27].

**Hypothesis 4.**  *Basic psychology needs satisfaction and learning engagement play a chain mediating role between perceived supervisor support and postgraduates’ academic procrastination*.

### 1.5. The Present Research

**Figure 1:**
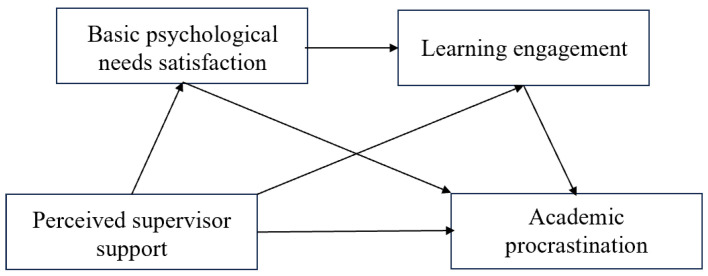
Hypothesis model diagram.

Currently, studies on the effects of teacher support on students’ academic procrastination have mainly focused on primary and secondary school students and undergraduate groups, and relatively little has been explored in the group of postgraduates [3,15]. Furthermore, most of the existing studies have focused on solely exploring the effects of internal or external factors on academic procrastination, and there is a lack of research on the interactions between the two, which means it is necessary to examine the potential mechanisms between perceived supervisor support and academic procrastination of postgraduate students by combining both internal and external influencing factors. Based on this, we proposed four hypotheses and a model diagram (see Figure 1):

## 2. Methods

### 2.1. Participants and Procedure

In October 2023, a convenience sampling survey was conducted through the Questionnaire Star platform. The survey targeted current postgraduate students across the country, and a total of 510 questionnaires were collected. During the subsequent data processing, we removed the questionnaires with unusually short or excessive response times, as well as the questionnaires with incorrect answers to the polygraph questions. As a result, we retained 448 valid questionnaires: questionnaires were received with a coefficient of with 87.8%, divided by gender, with 193 male students (43.1%) and 255 female students (56.9%), as well as divided by majors, with 167 students in the science and engineering category (37.3%), and 281 in the humanities and social sciences category (62.7%).

### 2.2. Measures

#### 2.2.1. Perceived Supervisor Support

This study revised the Perceived Supervisor Support Scale, which was an adaptation of the Interpersonal Behaviors Questionnaire (IBQ) developed by Rocchi et al., taking into account the specific circumstances of domestic postgraduate students and including certain items from the Teacher Autonomy Support, Structure, and Involvement Questionnaires developed by Connell and Wellborn (1991). This revised scale aimed to assess the degree of supervisor support perceived by postgraduate students throughout their interactions with their supervisors. Based on the results of the verification factor analysis, 6 items were deleted, and a total of 26 items were inclued. It contained two subscales: perceived supervisor support (15 items) and perceived supervisor thwarting (11 items), whereas perceived supervisor support included three sub-dimensions: autonomy support (4 items, e.g., “My supervisor supports my decisions”), competence support (7 items, e.g., “My supervisor encourages me to improve my skills”), and relationship support (4 items, e.g., “My supervisor takes the time to get to know me”). Perceived supervisor thwarting is divided into three sub-dimensions: autonomy thwarting (4 items, e.g., “My supervisor pressures me to do things their way”), competence thwarting (4 items, e.g., “My supervisor doubts my capacity to improve”), and relationship thwarting (3 items, e.g., “My supervisor does not care about me”), with the perceived supervisor thwarting subscale being reverse scored. The revised scale had an excellent fit (χ^2^ = 767.146, *df* = 284, χ^2^*/df* = 2.7, *RMSEA* = 0.062, *CFI* = 0.922, *TLI* = 0.911). The scale adopted a 5-point Likert scale ranging from 1 (“*not true at all of me*”) to 5 (“*very true of me*”), with higher scores indicating a higher degree of perceived supervisor support for postgraduate students. In this study, the Cronbach’s α coefficient of the scale was 0.944.

#### 2.2.2. Academic Procrastination

The Academic Procrastination Scale for postgraduate students revised by Hu (2008) was developed based on the Procrastination Assessment Scale-Students (PASS) designed by Solomon and Rothblum. It consists of two sections; in this study, only the first section of the scale was used, which includes six learning activities (e.g., “Read academic literature”), and under each learning activity, three questions were set up, assessing the level of procrastination, the extent to which procrastination was a disruptive problem, and the expectation of reducing procrastination. The scale was based on a 5-point Likert scale from 1 (“*never*”) to 5 (“*always*”), and the scores of 4 or 5 for the three items under each learning activity were defined as high degree of procrastination, high procrastination problem, and high expectation of reducing procrastination [35]. In this study, the Cronbach’s α coefficient of the scale was 0.939, and the scale had an acceptable fit (χ^2^ = 34.604, *df* = 9, χ^2^*/df* = 3.84, *RMSEA* = 0.08, *CFI* = 0.98, *TLI* = 0.967).

#### 2.2.3. Basic Psychological Needs Satisfaction

Postgraduate students’ basic psychological needs satisfaction was measured by The Basic Psychological Needs Scales (BPNS) developed by Gagne et al. (2003). This scale aims to assess the satisfaction degree of individual psychological needs [36]. The scale consists of 21 items, including three dimensions: autonomy needs (e.g., “I can make my own decisions in life”), competence needs (e.g., “For new skills and new knowledge, I am confident to learn well”), and relatedness needs (e.g., “I can get along well with my friends around me”), with seven items in each. Among them, nine items (3, 4, 7, 11, 15, 16, 18, 19, 20) are scored in reverse. The scale uses a 5-point Likert scoring ranging from 1 (“*not true at all of me*”) to 5 (“*very true of me*”). The higher the score, the higher degree of basic psychological needs satisfaction. In this study, the Cronbach’s α coefficient of this scale was 0.898, and the scale had an acceptable fit (χ^2^ = 516.802, *df* = 174, χ^2^*/df* = 2.97, *RMSEA* = 0.066, *CFI* = 0.904, *TLI* = 0.884).

#### 2.2.4. Learning Engagement

Postgraduate students’ learning engagement was measured by a modified version of the Utrecht Work Engagement Scale-Student (UWES-S) developed by Schaufeli et al.; this version was revised by Fang et al. (2008). It is mainly used to assess the degree of students’ engagement in learning [37]. The scale consists of 17 items, including three dimensions of vigor (6 items, e.g., “ When I study, I feel energetic”), dedication (5 items, e.g., “ I find study very meaningful”), and absorption (6 items, e.g., “ When I am studying, I feel that time passes quickly”), and it uses a 5-point Likert scale ranging from 1 (“*not true at all of me*”) to 5 (“*very true of me*”), with higher scores indicating higher degree of engagement with learning. The Cronbach’s alpha coefficient for this scale in this study was 0.943, and the scale had an acceptable fit (χ^2^ = 360.648, *df* = 106, χ^2^*/df* = 3.4, *RMSEA* = 0.073, *CFI* = 0.906, *TLI* = 0.879).

### 2.3. Data Analysis

We used SPSS 27.0 (IBM, Chicago, IL, USA) to calculate the descriptive analysis of the variables, including mean, standard deviation, and Pearson correlation. In addition, a structural equation model was constructed with perceived supervisor support as the independent variable and academic procrastination of postgraduate students as the dependent variable. The research hypothesis model was tested using Mplus 8.0 utilizing the Bootstrap method, with 5000 repeated sampling.

## 3. Results

### 3.1. Common Method Bias

We used a questionnaire as the means of gathering data, and there may have been common method bias. Therefore, the Harman single factor test was used to test the common method bias effect. The results showed that there were 12 common factors with eigenvalues greater than 1, among which the variance explained by the first common factor was 30.65%, less than 40%. It showed that there was no serious common method bias in the data of this study [38].

### 3.2. Descriptive Statistics and Correlation Analysis

As shown in Table 1, perceived supervisor support was significantly positively correlated with basic psychological needs satisfaction (*r* = 0.598, *p* < 0.001) and learning engagement (*r* = 0.503, *p* < 0.001), and it was significantly negatively correlated with academic procrastination (*r* = −0.389, *p* < 0.001); basic psychological needs satisfaction and learning engagement (*r* = 0.645, *p* < 0.001) were significantly positively correlated with academic procrastination (*r* = 0.488, *p* < 0.001) and significantly negatively correlated with academic engagement (*r* = −0.524, *p* < 0.001); and academic engagement was significantly negatively correlated with academic procrastination (*r* = −0.524, *p* < 0.001).

### 3.3. Examing the Mediation Model

We used a structural equation modeling approach to further assess the chain-mediated effects of basic psychological need satisfaction and learning engagement between perceived supervisor support and postgraduate students’ academic procrastination. The model was saturated, so fit indices were not presented here [39].

**Figure 2:**
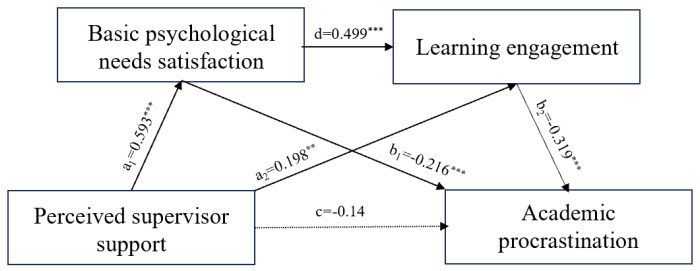
Chain mediation effect diagram.

The specific path coefficients were shown in Figure 2: perceived supervisor support was not significant in predicting academic procrastination of postgraduate students (*β* = −0.41, *p* > 0.05); perceived supervisor support significantly and positively predicted basic psychological need satisfaction of postgraduate students (*β* = 0.593, *p* < 0.001) and significantly and positively predicted learning engagement of postgraduate students (*β* = 0.198, *p* < 0.01); basic psychological needs satisfaction significantly positively predicted learning engagement (*β* = 0.499, *p* < 0.001) and significantly negatively predicted academic procrastination (*β* = −0.216, *p* < 0.001) of postgraduate students; and learning engagement significantly negatively predicted academic procrastination (*β* = −0.319, *p* < 0.001) of postgraduate students.

The results of the analyses of the mediating effect indicated (Table 2) that perceived supervisor support could influence the academic procrastination of postgraduate students through the direct effects of basic psychological need satisfaction and learning engagement, as well as through the chain-mediating effect of basic psychological need satisfaction → learning engagement. The mediating effect consisted of three paths indirect paths: (1) perceived supervisor support → basic psychological need satisfaction → academic procrastination, and the indirect effect of perceived supervisor support on academic procrastination via the mediation of basic psychological need satisfaction was significant [effect = −0.185, 95%CI (−0.302, −0.081)], with hypothesis 1 and 2 verified; (2) perceived supervisor support → learning engagement → academic procrastination, where the indirect effect of perceived supervisor support on academic procrastination via the mediation of learning engagement was significant [effect= −0.091, 95%CI (−0.167, −0.034)] and hypothesis 3 was verified; (3) perceived supervisor support → basic psychological need satisfaction → learning engagement → academic procrastination, where the indirect effect of perceived supervisor support on academic procrastination via the mediation of basic psychological need satisfaction to learning engagement was significant [effect = −0.136, 95%CI (−0.212, −0.081)] and hypothesis 4 was verified. Between perceived supervisor support and academic procrastination of postgraduate students, the value of the total mediating effect was −0.412, which accounted for 74.6% of the total effect (−0.552).

## 4. Discussion

Academic procrastination not only negatively affects the academic performance of postgraduate students but also causes serious damage to their mental health. First, academic procrastination can lead to a decline in the academic achievement of postgraduate students. Procrastination prevents students from completing their academic tasks on time, which directly affects their research progress, resulting in poor academic papers and even delayed graduation [8,10,40]. Second, academic procrastination can also adversely affect the mental health of graduate students. Procrastination is often accompanied by a decrease in anxiety, depression, and self-efficacy [4,10,40]. The deterioration of this mental state will not only affect their learning efficiency but also have a long-term impact on their overall well-being, and the long-term psychological burden may lead to students’ escape behavior [6,41] and further aggravate their procrastination problem, forming a vicious circle. Therefore, the problem of reducing academic procrastination for postgraduate students cannot be ignored.

### 4.1. The Influence of Perceived Supervisor Support on the Academic Procrastination of Postgraduate Students

This study explored the association between perceived supervisor support and academic procrastination in postgraduate students. The analysis results showed that perceived supervisor support could significantly negatively predict academic procrastination; this finding is consistent with the results of previous studies [3,10,15], verifying hypothesis 1. In other words, the more support postgraduate students perceived from their supervisors, the less they procrastinated in academic tasks. According to SDT, a supportive social environment plays a crucial role in stimulating and maintaining an individual’s intrinsic motivation and promoting an individual’s self-growth and mental health [42,43]. In the academic career of postgraduate students, they often face heavy academic tasks and pressure. Studies have shown that when students felt supportive resources and care from supervisors, they were more likely to transform these external supports into intrinsic learning motivation, enhancing students’ self-efficacy, that is, individuals’ confidence in their ability to complete specific tasks [44], which made students more proactive in completing academic tasks. It could improve learning efficiency and academic achievement, as well as reduce procrastination. Therefore, supervisors should establish a supportive teacher-student relationship with students [45], providing a safe learning environment so that students understand that they will not suffer undue criticism or accusations during the academic research process.

### 4.2. Basic Psychological Needs Satisfaction and Learning Engagemet Between Perceived Supervisor Support and the Academic Procrastination of Postgraduate Students

This study showed that perceived supervisor support not only directly affected the academic procrastination of postgraduate students, but that it also did so through three indirect paths.

First of all, perceived supervisor support can indirectly affect academic procrastination by satisfying the basic psychological needs of postgraduate students. The higher the degree of satisfaction of basic psychological needs, the lower the degree of academic procrastination. In the context of education, SDT believes that teachers can provide an environment that supports students’ basic psychological needs and positively influences students’ behaviors [42,43,46]. Based on SDT, many researchers have explained how teachers can construct teaching environments that support students’ needs for autonomy, competence, and relatedness, such as providing choices and meaningful reasons for students’ learning activities during guiding, affirming students’ thoughts and feelings, and reducing stress and control (autonomous support); providing constructive feedback that affirms students’ abilities (competency support); and showing warmth, care, and respect to students (relationship support) [13]. Positive results, like increased academic achievement and learning engagement [12], can be encouraged when teachers satisfy their students’ needs; conversely, when teachers obstruct their needs, positive outcomes can also be encouraged. Students’ academic maladjustment (procrastination, disengagement, etc.) will result from it [3,20,34]. The results of this study on the group of postgraduate students were consistent with previous studies.

Second, perceived supervisor support can also influence academic procrastination through the mediating role of learning engagement. During the study period of postgraduate students, supervisors play an important role, and their support can directly affect students’ learning engagement. When students perceive the support and encouragement from the supervisor, they will generate positive emotion and attitude, which will increase their learning engagement [31]. Enhanced engagement in learning means that students are more inclined to complete academic tasks on time rather than delaying or avoiding them [23]. 

Finally, this study also found that perceived supervisor support indirectly affects the academic procrastination of postgraduate students through the chain mediating of basic psychological need satisfaction and learning engagement. Supervisor support and encouragement will make students feel concerned, recognized, and understood, thus improving basic psychological needs satisfaction [34]. When basic psychological needs are met, postgraduate students will be more confident and more active in facing learning challenges, improve learning engagement, and manage their time more effectively. This will improve learning efficiency and reduce academic procrastination.

To sum up, we believe that in order to promote the academic development of postgraduate students, supervisors should pay attention to meeting the basic psychological needs of postgraduate students, including the support of autonomy, the cultivation of a sense of competence, and the establishment of a sense of relatedness. Specifically, supervisors can satisfy the basic psychological needs of students in the following ways: First, the supervisor should respect the wishes and choices of the students and make the students feel that their decisions and actions have a substantial impact on the research. Secondly, supervisors can provide students with challenging tasks and provide appropriate support and guidance when they encounter difficulties, helping students to overcome difficulties and enhance their self-confidence and sense of competence. Third, supervisors should establish a good relationship with students, pay attention to their life and study, let students feel cared for and supported, and encourage cooperation and communication between students to promote each other’s growth and common progress. By meeting the basic psychological needs of students, a positive learning atmosphere can be created, so as to allow research to continue more effectively and to lead to a decline academic procrastination.

## 5. Implications, Limitations, and Future Research Directions

First of all, this study revised the perceived supervisor support scale for postgraduate students in the domestic educational background, making up for the lack of a scale specifically designed to measure postgraduate students and also providing measurement tools for subsequent related research. Secondly, on the basis of previous studies, this study explored the mechanism of perceived supervisor support on academic procrastination for postgraduate students, making up for the lack of discussion on postgraduate students in previous studies that was based on the classroom environment for primary and secondary schools and undergraduate students, as well as providing a theoretical foundation for how supervisors should reduce the academic procrastination behavior of postgraduate students. At the same time, the research results had a certain guiding effect on how postgraduate supervisors get along and communicate with students, and it can help postgraduate supervisors further understand the reasons for students’ academic procrastination, so as to improve the learning engagement of postgraduate students, reduce academic procrastination, and improve the training quality of postgraduate students. 

There are also some shortcomings in this study: firstly, the limitations of the measurement tool mean that future research could further refine the revision of the psychometric scale by expanding the sample size and supplementing the interview method. Secondly, although this study explored the perspective of postgraduate students’ perceived supervisor behavior, supervisor–student interaction is a two-way process, and future research could collect data from supervisors in order to make the findings more comprehensive and provide more valuable practical advice for higher education.

## 6. Conclusions

(1) Perceived supervisor support, basic psychological needs satisfaction, and learning engagement are significantly correlated with academic procrastination of postgraduate students.
(2) Basic psychological needs satisfaction has an independent mediating effect between perceived supervisor support and the academic procrastination of postgraduate students.
(3) Learning engagement has an independent mediating effect between perceived supervisor support and the academic procrastination of postgraduate students.
(4) Basic psychological needs satisfaction and learning engagement play a chain mediating role between perceived supervisor support and the academic procrastination of postgraduate students.

## Figures and Tables

**Table 1:** Descriptive statistics and correlation analysis between variables (*n* = 448).

	*M ± SD*	1	2	3	4
1. Perceived supervisor support	106.14 ± 15.24	1			
2. Basic psychological needs satisfaction	81.05 ± 11.32	0.598 ***	1		
3. Learning engagement	62.08 ± 12.31	0.503 ***	0.645 ***	1	
4. Academic procrastination	15.13 ± 5.08	−0.389 ***	−0.488 ***	−0.524 ***	1

*** *p* < 0.001

**Table 2:** Analysis of the mediating effect between perceived supervisor support, basic psychological needs satisfaction, learning engagement, and academic procrastination.

Paths	Effect	Percentage of Total Effect	95% Confidence Interval	
			Lower	Upper
PSS→BPNS→AP	−0.185 **	0.34	−0.302	−0.081
PSS→LE→AP	−0.091 **	0.16	−0.167	−0.034
PSS→BPNS→LE→AP	−0.136 ***	0.25	−0.212	−0.081
Total effect	−0.552 ***		−0.676	−0.431

PTS, perceived supervisor support; BPNS, basic psychological need satisfaction; LE, learning engagement; AP, academic procrastination. ** *p* < 0.01, *** *p* < 0.001.

## Data Availability

The data presented in this study are available from the corresponding author upon request.
